# FABP7: A Regulator of Neuro-Immune Metabolic Networks and Therapeutic Vulnerabilities in Glioma

**DOI:** 10.3390/cancers18132029

**Published:** 2026-06-23

**Authors:** Yool Lee, Yeena Kee, Sukanya Bhoumik, Carlos C. Flores, Jorge Zepeda-Reyes, Dylan A. Nasinec, Peyton Burpee, Monte Schell, Yuji Owada, Jason R. Gerstner

**Affiliations:** 1 Department of Translational Medicine and Physiology, Elson S. Floyd College of Medicine, Washington State University, Spokane, WA 99202, USA; yeena.kee@wsu.edu (Y.K.); sukanya.bhoumik@wsu.edu (S.B.); carlos.c.flores@wsu.edu (C.C.F.); jorge.zepedareyes@wsu.edu (J.Z.-R.); dylan.nasinec@wsu.edu (D.A.N.); peyton.burpee@wsu.edu (P.B.); monte.schell@wsu.edu (M.S.); 2 Department of Integrative Physiology and Neuroscience, College of Veterinary Medicine, Washington State University, Pullman, WA 99164, USA; 3 Sleep and Performance Research Center, Washington State University, Spokane, WA 99202, USA; 4 Steve Gleason Institute for Neuroscience, Washington State University, Spokane, WA 99202, USA; 5 Department of Organ Anatomy, Graduate School of Medicine, Tohoku University, Seiryo-cho 2-1, Aobaku, Sendai 980-8575, Japan; owada@med.tohoku.ac.jp

**Keywords:** FABP7, glioma, tumor microenvironment, lipid metabolism, post-transcriptional regulation, tumor microtubes, cancer immunotherapy

## Abstract

Fatty acid-binding protein 7 (FABP7) is a multifunctional lipid chaperone that is enriched in radial glia and astrocytes within the central nervous system (CNS) and is frequently upregulated in glioma. Beyond its established roles in glial development, lipid homeostasis, and circadian regulation, growing evidence positions FABP7 at the intersection of tumor metabolism, neuronal activity, and immune modulation in the brain. In this review, we integrate the physiological functions of FABP7 in glial cells with its tumor-intrinsic and microenvironmental roles in glioma. We summarize how gliomas co-opt FABP7-dependent metabolic, transcriptional, and post-transcriptional programs to promote stemness, lipid remodeling (e.g., altered fatty acid composition, lipid droplet formation, and lipid peroxidation resistance), inflammatory signaling, and invasive growth, including nuclear FABP7-mediated transcriptional activation linked to oncogene status. Furthermore, we discuss the role of FABP7 in shaping the tumor–neuro–immune interface, including regulating immunosuppressive gene networks, pro-tumoral macrophage polarization, resistance to T-cell-induced ferroptosis and immunotherapy, and tumor microtube-mediated integration into neuronal circuits to support glioma progression. Finally, we highlight therapeutic opportunities and challenges, including small-molecule FABP7 inhibitors, brain-directed delivery strategies, chronotherapeutic considerations, and combination approaches with immunotherapy. Collectively, this work positions FABP7-centered metabolic, circadian, and neuro-immune networks as potential vulnerabilities in glioma, linking fundamental glial biology to glioma therapeutics.

## Simple Summary

Gliomas are highly aggressive primary brain tumors characterized by metabolic reprogramming, resistance to therapy, and suppression of anti-tumor immune responses. Emerging evidence indicates that fatty acid-binding protein 7 (FABP7), a brain-enriched lipid chaperone involved in glial development, lipid metabolism, and circadian regulation, is aberrantly upregulated in glioma. In this review, we examine how FABP7 contributes to tumor progression by promoting metabolic adaptability, stem-like properties, resistance to oxidative stress, immune evasion, and interactions between tumor cells and neuronal networks. We aim to integrate current knowledge of FABP7-driven metabolic and immune pathways and to identify potential therapeutic vulnerabilities. A clearer understanding of FABP7-centered networks may inform the development of combined metabolic and immunotherapeutic strategies and provide a framework for targeting tumor–brain microenvironment interactions in glioma.

## 1. Introduction

Gliomas are a heterogeneous group of primary brain tumors that occur in both children and adults and arise from glial lineage cells, including oligodendrocyte-like and astrocyte-like cells, within the central nervous system (CNS) [1]. In recent years, the World Health Organization (WHO) has classified gliomas into grades I to IV [2,3], with the 2021 classification emphasizing genetic differences by distinguishing between grade IV astrocytomas and isocitrate dehydrogenase (IDH)-wild-type glioblastomas [3]. Furthermore, in The Cancer Genome Atlas (TCGA) database, grade II and III tumors are lower-grade gliomas (LGGs), while grade IV tumors are classified as glioblastomas (GBMs). Despite significant advances in surgery, chemotherapy, and radiotherapy, glioma prognosis remains poor, with a median survival of 1.25 years for GBM patients and 6.5–8 years for LGG patients [4,5,6].

Gliomas exhibit profound metabolic reprogramming and immunosuppressive tumor microenvironments that blunt immunotherapy responses [7,8,9]; therefore, understanding the molecular regulators that intertwine metabolism, gene regulation, and immune interactions in these cancers is vital for new therapeutic approaches. Primary brain tumors and brain metastases exhibit a strong dependence on lipid metabolism, including enhanced de novo lipogenesis, fatty acid uptake, and lipid droplet (LD) accumulation. In glioblastoma (GBM), key lipogenic enzymes such as fatty acid synthase (FASN), ATP citrate lyase (ACLY), and acetyl-CoA carboxylase (ACC) are upregulated through Epidermal Growth Factor Receptor (EGFR)/mechanistic Target of Rapamycin (mTOR)–Sterol Regulatory Element-Binding Protein 1 (SREBP-1) signaling, promoting tumor growth and stemness [10,11]. This lipogenic program drives extensive LD accumulation, which serves as an energy reservoir supporting tumor survival under metabolic stress and is particularly enriched in hypoxic regions and glioma stem cell niches [12,13]. Beyond tumor-intrinsic functions, LDs contribute to immune evasion by promoting the formation of lipid-laden tumor-associated macrophages that enhance angiogenesis, mesenchymal transition, and tumor progression while impairing antitumor immunity [14]. Lipid metabolism also supports cholesterol recycling between macrophages and GBM cells and promotes ferroptosis resistance [15,16]. Similar metabolic adaptations occur in brain metastases, where cancer cells rely on FASN-dependent lipogenesis to survive within the lipid-poor brain microenvironment, and FASN inhibition suppresses metastatic growth [17]. Collectively, these findings identify dysregulated lipid metabolism and LD accumulation as key hallmarks of primary and metastatic brain tumors that cooperate with lipid transport pathways, including those mediated by FABP7, to sustain tumor stemness, immune evasion, and disease progression.

Fatty acid-binding protein 7 (FABP7), also known as brain lipid-binding protein (BLBP) or brain-type fatty acid-binding protein (B-FABP), is a ~15 kDa brain-enriched protein that is highly expressed in glial cells and neural stem/progenitor cells (NSCs/NPCs) throughout the CNS [18,19,20]. As a lipid chaperone, FABP7 binds and traffics long-chain polyunsaturated fatty acids (PUFAs) to specific subcellular compartments, thereby regulating fatty acid uptake, metabolism, storage and influencing gene expression, development, sleep and circadian rhythms, inflammatory signaling, and overall brain homeostasis (recently reviewed by our group [21]). In cancers, FABP7 is frequently upregulated (up to 20-fold in some tumors) and is generally associated with worse patient prognosis [22]. GBM is no exception, with FABP7 being overexpressed in a majority of GBM tumors, particularly in stem-like cells and invasive cell subpopulations, and its expression levels tending to increase with tumor grade [23]. Despite substantial evidence supporting a pro-tumorigenic role for FABP7 in glioma, its biological and clinical significance appears to be highly context dependent. For example, FABP7 function is likely lipid- and microenvironment-dependent, since docosahexaenoic acid (DHA)-bound FABP7 can suppress glioma cell migration, whereas arachidonic acid (AA)-bound FABP7 promotes migration through pro-inflammatory lipid signaling [24,25]. Moreover, recent analyses indicate that FABP7-associated immune and prognostic signatures are more pronounced in lower-grade glioma than in GBM, suggesting differential roles across glioma subtypes [26]. Thus, this evidence indicates that FABP7 may function as a context-dependent lipid signaling regulator whose effects are shaped by ligand availability, molecular subtype, and tumor grade.

Although FABP7 is the most extensively studied FABP isoform in glioma biology, it is important to recognize that the nine-member FABP family, encompassing FABP1 through FABP9, each named for the tissue in which it was first identified, shares a conserved beta-barrel tertiary structure and broad function as intracellular lipid chaperones, yet exhibits distinct ligand preferences, tissue distributions, and downstream signaling outputs that confer isoform-specific biological roles [19]. Among the brain-expressed isoforms, FABP3 (heart-type), FABP5 (epidermal-type), FABP6 (ileal-type), and FABP7 (brain-type) are all detectable in glioma tissues, yet their functional contributions are mechanistically distinct. FABP3 and FABP7 both promote LD accumulation under hypoxic conditions and preferentially bind long-chain PUFAs, but FABP3 favors ω-6 arachidonic acid (AA) whereas FABP7 exhibits substantially higher affinity for ω-3 docosahexaenoic acid (DHA), a difference with direct consequences for tumor cell migration and signaling [25,27,28]. FABP5, which preferentially binds saturated and monounsaturated fatty acids, is upregulated in both GBM and lower-grade gliomas, where it promotes proliferation, invasion, temozolomide resistance, and epithelial–mesenchymal transition (EMT) via canonical NF-κB signaling, a mechanism mechanistically divergent from FABP7’s PUFA trafficking and nuclear gene regulatory programs [29,30]. FABP6, a bile acid carrier protein atypically expressed in glioma, promotes invasion and angiogenesis through upregulation of matrix metalloproteinase-2 (MMP-2) and vascular endothelial growth factor (VEGF) expression through extracellular signal-regulated kinase (ERK) and c-Jun N-terminal kinase (JNK) signaling [31]. What distinguishes FABP7 from these overlapping isoforms is a unique convergence of features: its exceptionally high enrichment in radial glia-like NSCs/NPCs and GSC subpopulations, its capacity for DHA-dependent nuclear translocation, a behavior not shared by FABP3 or FABP5, thereby directly coupling lipid sensing to transcriptional regulation, and its coordinate roles in orchestrating oncogenic signaling processes in glioma [22,32].

At the molecular and cellular level, FABP7 is predominantly cytoplasmic and promotes fatty acid uptake, lipid droplet formation, and oncogenic signaling, while its nuclear localization is strongly associated with aggressive glioma subtypes, including epidermal growth factor receptor (EGFR)-amplified and IDH1-wild-type glioblastomas [26,33,34]. Furthermore, beyond its tumor-intrinsic functions, recent studies have shown that FABP7 is enriched in malignant and glial-lineage cells and drives onco-metabolic and immunosuppressive gene programs that correlate with poor patient prognosis [26]. Elevated FABP7 expression correlates with increased infiltration of regulatory T-cells and other suppressive immune populations, reduced CD8^+^ T-cell presence, enhanced pro-tumorigenic M2 macrophage polarization, and resistance to T-cell-mediated ferroptosis, collectively promoting immune evasion and resistance to immune checkpoint-mediated killing [35,36,37]. In addition, astrocytic FABP7 is critical for dendritic arborization, excitatory synapse formation, and synaptic transmission [38], processes linked not only to neurological disorders such as schizophrenia [39,40,41] and epilepsy [42,43]. These processes are also linked to glioma progression [44,45], where FABP7-enriched tumor microtubes may facilitate tumor–neuron connectivity, network hyperexcitability, and invasive growth [46].

In this review, we integrate the physiological and pathological roles of FABP7 by highlighting its functions in glial lipid trafficking and metabolism, circadian regulation, and neuroinflammatory signaling, and how these programs are co-opted in glioma to promote stemness, metabolic adaptation, immune evasion, and tumor–neuron integration. We further discuss FABP7-centered metabolic–neuro–immune networks in glioma that can be targeted using various therapeutic strategies, including FABP7 inhibitors, targeted delivery approaches, and combination immunotherapy.

## 2. Neuro-Physiological and Pathological Roles of FABP7 in Glial Cells

### 2.1. FABP7 Regulates Neurodevelopment and Glial Lipid Homeostasis Through Fatty Acid Trafficking

**Figure 1:**
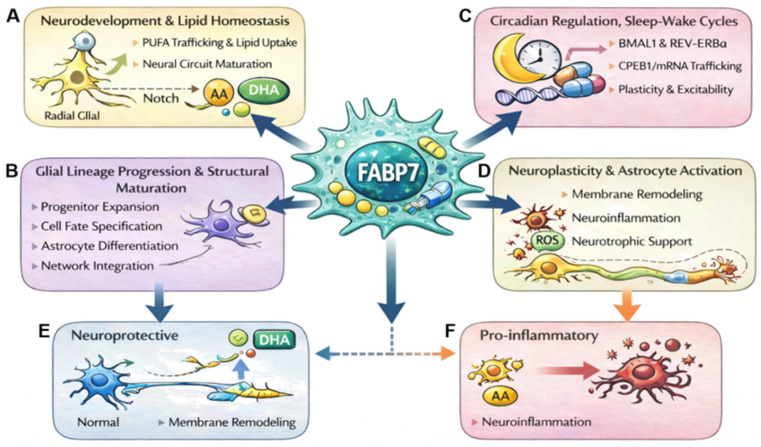
Regulatory Functions of FABP7 in Glial Physiology and Pathological States. Schematic overview illustrating the multifunctional roles of fatty acid-binding protein 7 (FABP7) in glial biology under physiological and pathological conditions. Center: FABP7-expressing glial cell. (**A**) Left (upper): During neurodevelopment, FABP7 regulates lipid homeostasis by mediating polyunsaturated fatty acid (PUFA) trafficking, membrane synthesis, and neural circuit maturation, acting downstream of Notch signaling in radial glial cells. (**B**) Left (middle): FABP7 supports neurogenesis and glial differentiation through lipid-dependent mechanisms that influence progenitor maintenance and maturation. (**C**) Right (upper): FABP7 functions as a circadian ‘clock-controlled gene’ in astrocytes, with rhythmic expression regulated by core clock components such as BMAL1 and REV-ERBα, and purported activity-dependent mRNA trafficking mediated by cytoplasmic polyadenylation element binding protein 1 (CPEB1), thereby linking metabolism to sleep–wake cycles and neuronal excitability. (**D**) Right (middle): Under pathological conditions, FABP7 contributes to neuroplasticity and astrocyte activation, promoting membrane remodeling, inflammatory signaling, and reactive oxygen species (ROS) responses. (**E**) Bottom (left): Ligand-dependent interactions with docosahexaenoic acid (DHA) support neuroprotective and anti-inflammatory pathways in homeostatic conditions. (**F**) Bottom (right): Association with arachidonic acid (AA) promotes pro-inflammatory signaling and neurotoxic astrocyte phenotypes under disease states.

Physiologically, FABP7 plays a pivotal role in the developing brain by coordinating lipid metabolism with neural progenitor maintenance, glial differentiation, and neural circuit maturation [21] (Figure 1).

FABP7 is expressed in both the cytoplasm and nucleus of immature astrocytes and radial glial cells, which are neural progenitors that generate neurons and astrocytes [47,48,49]. In radial glial cells, FABP7 has been identified as a downstream target of Notch signaling, a pathway that maintains progenitor identity, promotes proliferative signaling during early neurogenesis, and regulates the timing of differentiation [48]. Furthermore, by chaperoning PUFAs, like arachidonic acid (AA) and docosahexaenoic acid (DHA), FABP7 influences membrane synthesis and energy utilization during neurodevelopment [50,51,52]. In accordance with these functions, mice lacking *Fabp7* exhibit defects in neurogenesis and astrocyte maturation [48,53]. Loss of *Fabp7* also results in disrupted dendritic architecture and reduced spine density of astrocytes and cortical pyramidal neurons, which is accompanied by synaptic deficits in the medial prefrontal cortex, including a reduced number of excitatory synapses and decreased frequency and amplitude of miniature excitatory postsynaptic currents [38]. A recent study further showed that FABP7 is required for optimal differentiation of oligodendrocyte progenitor cells (OPCs) during myelination [54]. This process is heavily dependent on lipid synthesis, as the myelin sheath is composed primarily of fatty acid-rich lipids [54]. Notably, this myelination delay was transient, with full myelination established before adulthood, and FABP7 was dispensable for remyelination, suggesting that therapeutic FABP7 inhibition in adults may have a limited impact on myelin homeostasis [54]. In mature astrocytes, FABP7 regulates lipid homeostasis by facilitating the trafficking of long-chain fatty acids into lipid droplets (LDs), which serve as both storage depots and substrates for mitochondrial β-oxidation, thereby protecting astrocytes against reactive oxygen species (ROS)-induced toxicity [55]. Overall, FABP7 acts as a lipid sensor and distributor, enabling glial cells to adapt their membranes, fuel usage, and signaling in response to developmental and environmental cues.

### 2.2. FABP7 Functions as a “Glial Clock” Integrator, Coupling Circadian Rhythms to Astrocyte Metabolism and Sleep–Wake Behavior

An important aspect of FABP7 biology is its integration with circadian signaling in glial cells [21]. In mammals, circadian rhythms are governed by a cell-autonomous transcription–translation feedback loop in which the transcriptional activators brain and muscle Arnt-like 1 (BMAL1; also known as aryl hydrocarbon receptor nuclear translocator-like protein 1, ARNTL) and circadian locomotor output cycles kaput (CLOCK) drive the rhythmic expression of their repressors, the period (PER1, PER2, and PER3) and cryptochrome (CRY1 and CRY2) circadian regulator proteins [56,57]. This core molecular oscillator is further reinforced by an auxiliary regulatory loop in which nuclear receptor subfamily 1 group D members 1 and 2 (REV-ERBα and REV-ERBβ; NR1D1 and NR1D2) act as transcriptional repressors, while RAR-related orphan receptors alpha and beta (RORα and RORβ; RORA and RORB) function as transcriptional activators to fine-tune *BMAL1* expression and sustain robust circadian cycling [56,57]. FABP7 mRNA levels oscillate in a synchronized fashion over the 24 h light/dark cycle throughout the mammalian brain, including regions governing circadian rhythms and sleep–wake behavior [58,59]. This rhythmicity is driven by core clock mechanisms: REV-ERBα binds to ROR response elements (ROREs) in the *Fabp7* promoter to repress transcription at certain times [60], and *Bmal1* is required to maintain the normal amplitude of *Fabp7* expression [61,62]. Notably, *Fabp7* mRNAs are not only cyclically transcribed but also show circadian trafficking to peripheral astrocytic processes (PAPs), where they are thought to undergo local translation [63]. Gerstner et al. demonstrated that during the active (wake) phase, *Fabp7* mRNA and protein are enriched in PAPs, with their levels declining during the light period (sleep phase in nocturnal mice). Mechanistically, it was shown that cytoplasmic polyadenylation element binding protein 1 (CPEB1) binds to conserved cytoplasmic polyadenylation elements (CPEs) within the 3′ untranslated region of *Fabp7* mRNA to dynamically regulate CPE-mediated translation. CPE-mediated changes in poly(A) tail length can enhance transcript stability and translational efficiency, thereby enabling state-dependent, localized FABP7 protein synthesis at the tripartite synapse to support lipid trafficking and coupling of metabolism to neuronal activity [64]. It has also been shown that manipulating FABP7 alters sleep phenotypes across species, with systemic loss of FABP7 in flies and mice leading to fragmented or arrhythmic sleep, compared with wild-type controls [65,66,67], and genetic induction of murine Fabp7 or the *Drosophila* homolog, dFabp, augments memory and increases sleep in flies [65,68]. Beyond sleep, a reciprocal interaction of FABP7 with activity-dependent molecular processes is noteworthy. Neuronal stimulation, such as kainic acid-induced seizures, can increase astrocytic *Fabp7* mRNA expression in the hippocampal glia of adult rats [69], paralleling the induction of activity-dependent plasticity genes, such as brain-derived neurotrophic factor (*Bdnf*) [70]. Importantly, our recent work has further shown that astrocytic loss of *Fabp7* in mice lowers the seizure threshold in a time-of-day-dependent manner. This is also associated with broad alterations in transcriptional, proteasomal and mitochondrial protein networks, suggesting potential mechanistic links between FABP7-mediated circadian regulation, mRNA regulation, proteostasis, and neuronal excitability [71,72]. Together, these findings support FABP7 as a central “glial clock” integrator that couples astrocytic metabolism with pre- and post-transcriptional control to neuronal activity rhythms, which has broad implications for brain function in normal health and disease.

### 2.3. FABP7 Modulates Neuroplasticity and Drives Neurotoxic Astrocyte Activation in Neuroinflammatory and Neuropathological Conditions

Beyond its canonical role in lipid metabolism, FABP7 contributes broadly to brain physiology and pathology by coordinating astrocyte-dependent neuroplasticity, inflammatory signaling, cellular proliferation, and neuron–glia interactions that shape neural circuit function and vulnerability under both homeostatic and disease conditions. FABP7 has been implicated in lipid-mediated NMDA-dependent neurotransmission and cognitive behavior [39,65,67,68]. More recently, FABP7 has been shown to alleviate depression-like behaviors under chronic stress by regulating neuroinflammation and hippocampal spinogenesis [73]. In addition, astrocytic FABP7 has been shown to play important roles in neuroinflammatory responses and astrocyte activation. In the stab-injured cortex, *Fabp7*-deficient mice exhibit reduced numbers of reactive and proliferating astrocytes, and primary astrocytes lacking *Fabp7* show impaired proliferation, indicating that FABP7 supports astrocyte proliferation through the regulation of cellular fatty acid homeostasis [74]. Furthermore, recent studies have shown that FABP7 is upregulated in astrocytes under several pathological conditions, including Alzheimer’s disease (AD), where it is enriched around amyloid plaques in response to amyloid-β, amyotrophic lateral sclerosis (ALS), and lipopolysaccharide-induced endotoxemia [75,76,77]. In these contexts, FABP7 promotes a nuclear factor kappa-light-chain-enhancer of activated B-cells (NF-κB)-dependent pro-inflammatory program that drives astrocytes toward a neurotoxic phenotype and compromises neuronal survival [75,76,77]. Importantly, this inflammatory activity is dependent upon FABP7’s lipid-binding capacity, as FABP7 silencing or the expression of ligand-binding-deficient mutants attenuates astrocyte inflammatory activation and protects neurons from glia-mediated injury [75,76,77]. Intriguingly, FABP7 interacts with both AA and DHA [50], leading to divergent physiological outcomes [24,78]. Association with AA is linked to the activation of pro-inflammatory signaling and astrogliosis, potentially amplifying neuroinflammatory responses [24,27,75,78], whereas binding to DHA promotes anti-inflammatory pathways and contributes to neuroprotection [24,27]. In AD, this ligand-dependent functional duality highlights FABP7 as a context-dependent regulator of astrocyte inflammatory and metabolic states, suggesting that selective modulation of its lipid interactions may represent a promising strategy to rebalance metabolic homeostasis and attenuate neuroinflammatory pathology [79].

Collectively, through lipid transport and metabolism, gene regulation, dynamic mRNA handling, cell proliferation, and the regulation of inflammatory state under physiological and pathological conditions, FABP7 supports core astrocytic functions that, when co-opted or dysregulated, may create a permissive neural and immune microenvironment conducive to glioma initiation and progression.

## 3. FABP7 in Glioma: Tumor-Intrinsic Roles in Stemness, Metabolism, and Survival

### 3.1. FABP7 Drives Glioma Stem Cell Phenotypes Through Oncogenic Signaling Networks

Glioma cells frequently upregulate FABP7, reactivating a developmental program that grants them metabolic and survival advantages. FABP7-positive radial glia have been proposed as the cells-of-origin for certain malignant gliomas [80]. In fact, earlier studies have shown that high FABP7 expression correlates with enhanced proliferative and stem-like properties of glioma cells, with elevated expression in neurospheres compared to adherent cells and reduced proliferation and invasive migration upon FABP7 downregulation, consistent with the role of glioma stem-like cells (GSCs) in driving glioblastoma recurrence and aggressiveness [81]. This is further supported by gene expression analyses of patient tumors which found that FABP7 levels are elevated in subsets of GBM enriched for neural stem cell markers (e.g., Sox2) and aggressive behavior [23]. In addition, multiple studies have shown that FABP7 modulates several oncogenic signaling pathways, including those regulated by proto-oncogene tyrosine-protein kinase Src [82], mitogen-activated protein kinase kinase/extracellular signal-regulated kinase (MEK/ERK) [83,84], NF-κB [76], and Wnt/β-catenin [85,86], across diverse cancer types. These findings suggest that FABP7 could function as a broad activator of oncogenic networks that promote self-renewal and invasive capacity in glioma cells (Table 1).

### 3.2. Nuclear FABP7 Integrates Oncogene Status with Transcriptional Programs to Drive Glioma Progression

Accumulating evidence indicates that the expression level and subcellular localization of FABP7 are closely associated with the mutational status of key oncogenes in glioma, with important implications for tumor progression and clinical outcomes. Immunohistochemical analyses have demonstrated that nuclear rather than cytoplasmic localization of FABP7 correlates with EGFR amplification and enhanced tumor invasiveness, and is associated with poor prognosis in EGFR-overexpressing glioblastoma [87,88]. Mechanistically, FABP7’s lipid-binding function has been shown to mediate its nuclear localization and activate oncogenic signaling pathways through several interconnected mechanisms. Upon binding fatty acids (DHA, oleic acid), FABP7 undergoes conformational changes that expose its nuclear localization signal (NLS), facilitating its translocation to the nucleus. There, it can activate transcription factors such as peroxisome proliferator-activated receptor gamma (PPARγ), a lipid-sensing nuclear receptor that regulates genes involved in fatty acid metabolism, thereby linking intracellular lipid dynamics to transcriptional programs that shape cellular metabolic state [25,92]. In the nucleus, FABP7 also delivers fatty acids for triglyceride synthesis and nuclear LD assembly. These nuclear LDs colocalize with promyelocytic leukemia nuclear bodies (PML NBs), triggering epigenetic modifications that activate proliferation genes [89]. Recent studies further show that nuclear FABP7 is more abundant in IDH1-wild-type glioblastomas than in IDH1-mutant lower-grade gliomas, where it promotes tumor cell proliferation by epigenetically regulating caveolin-1 expression through interaction with ATP-citrate lyase (ACLY), a key enzyme in acetyl-CoA metabolism and caveolae formation [33,34]. Caveolae are specialized lipid raft-like membrane microdomains that scaffold oncogenic signaling complexes, regulate receptor trafficking and nutrient uptake, and enhance membrane adaptability, collectively supporting proliferative and metabolic demands in aggressive tumor cells [93]. More recently, FABP7 has also been shown to interact with the nuclear receptor retinoid X receptor-α (RXRα), activating a PUFA-dependent transcriptional program that induces cancer stem cell (CSC) and epithelial–mesenchymal transition (EMT) markers, including SRY (sex-determining region Y)-box transcription factor 2 (SOX2) and zinc finger E-box binding homeobox 1 (ZEB1) in GBM cell lines and patient-derived GSCs, thereby enhancing glioma stemness and invasiveness [90,94]. Collectively, these findings highlight nuclear FABP7 as a critical transcriptional regulator that integrates metabolic signaling with gene expression programs driving glioma progression.

## 4. FABP7 and Tumor–Neuro–Immune Crosstalk in the Glioma Microenvironment

### 4.1. FABP7 Orchestrates Immunosuppressive Gene Networks and Shapes “Cold” Glioma Microenvironments

**Figure 2:**
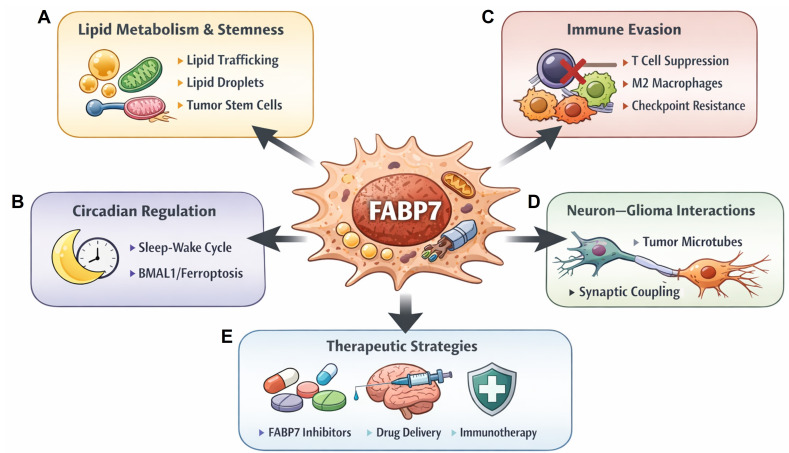
FABP7-centered neuro-immune-metabolic networks and therapeutic vulnerabilities in glioma. Schematic overview of how fatty acid-binding protein 7 (FABP7), a brain-enriched lipid chaperone expressed in astrocytes and neural progenitors, is repurposed in glioma to coordinate tumor-intrinsic programs and microenvironmental interactions. (**A**,**B**) Left (upper and middle): In glial cells, FABP7 regulates fatty acid (FA) trafficking, including polyunsaturated fatty acid handling, lipid droplet (LD) formation, and circadian rhythmicity. In glioma, tumor-intrinsic FABP7 promotes cancer stemness, metabolic reprogramming, oxidative stress tolerance, lipid droplet accumulation, ferroptosis resistance, and invasive growth. Notably, FABP7 enhances the transcription of ferroptosis-protective genes, including the core circadian regulator BMAL1 (Brain and Muscle ARNT-Like 1). Furthermore, nuclear localization of FABP7 has been associated with oncogene-dependent transcriptional programs that reinforce malignant phenotypes. (**C**) Right (upper): FABP7 is associated with immunosuppressive (“cold”) glioma states characterized by reduced cytotoxic T-cell activity, increased regulatory/suppressive immune populations, and pro-tumorigenic macrophage polarization, contributing to resistance to immune checkpoint-mediated killing and T-cell-induced ferroptosis. (**D**) Right (middle): FABP7 supports tumor–neuron crosstalk, with FABP7-enriched tumor microtubes (TMs) coupling glioma cells to neuronal networks, which may promote hyperexcitability/seizure susceptibility. (**E**) Bottom: Potential treatment strategies include small-molecule FABP7 inhibitors (e.g., MF6, SBFI-26), brain-directed delivery approaches, and combination regimens (e.g., with immune checkpoint blockade) to disrupt FABP7-driven metabolic and immune-evasive programs.

Cancer frequently establishes an immunosuppressive tumor microenvironment (TME) that dampens antitumor immune responses, thereby facilitating tumor progression and therapeutic resistance [95,96]. In addition to its tumor-intrinsic effects, growing evidence suggests that FABP7 also controls tumor-extrinsic functions, particularly those that modulate the glioma TME (Figure 2).

Using an integrated transcriptomic analysis, we recently showed that FABP7 mediates the expression of multiple “onco-immune” genes that collectively shape tumor immunity [26]. In that study, RNA-seq of *Fabp7*-KO mouse brains and FABP7-overexpressing (OV) human astrocytes revealed that altering FABP7 levels drives changes in pathways related to inflammation, extracellular matrix organization, and immune cell recruitment [26]. Notably, genes such as enolase 1 (*Eno1*), mucin 1 (*Muc1*), collagen type V alpha 1 chain (*Col5a1*), and interleukin 11 (*Il11*), which are known to contribute to a pro-tumorigenic and immunosuppressive microenvironment, were significantly downregulated in *Fabp7* KO brains but were upregulated in FABP7-OV human astrocytes [26]. Consistent with these observations, analyses of glioma patient datasets revealed that higher *FABP7* expression was associated with increased infiltration of immunosuppressive cell populations, including regulatory T-cells (Tregs), cancer-associated fibroblasts (CAFs), and myeloid-derived suppressor cells (MDSCs), alongside reduced infiltration of antitumor immune cells, such as CD8^+^ T-cells, CD4^+^ T-cells, and macrophages, within the TME [26]. Importantly, this immune landscape correlated with poorer clinical outcomes, particularly in patients with LGGs [26]. Thus, these data suggest that high levels of FABP7 may be a signature of “cold” glioma immune microenvironments, where tumor-promoting inflammation and suppressive immune cells predominate.

### 4.2. FABP7 Confers Ferroptosis Resistance and Promotes Immune Checkpoint Therapy Evasion

Beyond its role in promoting tumorigenic, immunosuppressive gene expression programs, emerging evidence is beginning to elucidate direct mechanistic links between FABP7 and immune modulation. A recent study by Freitas-Cortez et al. [36] used multiple cell lines, including programmed cell death protein 1 (PD-1)-sensitive and PD-1-resistant 344SQ lung adenocarcinoma derivatives, B16F10 murine melanoma cells, and QPP7 murine GBM cells, to show that FABP7 upregulation enables tumor cells to evade T-cell-induced ferroptosis, an iron-dependent lipid peroxidation-driven cell death pathway employed by cytotoxic T-cells to mediate antitumor immunity within the TME [97,98,99]. Mechanistically, FABP7 promotes ferroptosis resistance by reprogramming lipid metabolism via suppression of the pro-ferroptotic enzyme lysophosphatidylcholine acyltransferase 3 (LPCAT3), which incorporates PUFAs into membrane phospholipids and renders them susceptible to oxidation, and the enrichment of monounsaturated fatty acids (MUFAs) and triacylglycerols, which limit ROS generation and lipid peroxidation [36]. Notably, this ferroptosis-resistant phenotype intersects with circadian regulation, as FABP7 upregulates the core clock factor BMAL1, which exerts antioxidant and cytoprotective functions [36]. Functional inhibition of *Fabp7* via specific shRNA restored the susceptibility of tumor cells to immune-induced ferroptosis, restoring their susceptibility to immune-mediated killing [36]. Moreover, *Fabp7* was among the most highly upregulated genes in tumors resistant to PD-1 blockade, compared with PD-1-sensitive tumors, following treatment with PD-1 inhibitors [36]. Notably, combining Fabp7 inhibition with immune checkpoint blockade (ICB) targeting PD-1 and its ligand programmed death-ligand 1 (PD-L1) significantly enhanced antitumor immune responses and suppressed tumor growth in preclinical models [36]. Consistent with these findings, high *FABP7* expression is associated with reduced CD8^+^ T-cell infiltration and poorer survival outcomes, highlighting its potential utility as a biomarker for predicting immunotherapy response [36]. Collectively, these findings indicate that FABP7 coordinates metabolic adaptation, circadian signaling, and resistance to lipid peroxidation-driven cell death to promote immune evasion, supporting glioma survival and therapeutic resistance.

### 4.3. FABP7 Drives Macrophage M2 Polarization and Metabolic Reprogramming in the TME

It has been suggested that tumor-derived fatty acids promote immunosuppression by inducing the expansion of neutrophils and M2-type tumor-associated macrophages (TAMs) that support tumor progression and metastasis while suppressing antitumor immune responses [100,101,102,103]. A recent study in a liver cancer model reported that tumor-induced FABP7 upregulation in macrophages enhanced lipid uptake and M2 polarization and suppressed cytotoxic CD8^+^ T-cells via pleiotrophin (PTN) and phosphoinositide 3-kinase (PI3K)-AKT signaling [104], collectively promoting tumor immune evasion and creating a metabolic and immunosuppressive niche that facilitates cancer progression and therapeutic resistance [105,106]. Similarly, another recent study found that *Fabp7*-deficient hepatic macrophages exhibited impaired PPARγ activation and reduced expression of PPARγ target genes, including transforming growth factor-β (TGF-β) and chemotactic factors, which led to diminished macrophage M2 polarization and decreased recruitment of CD4^+^ T-cells into fibrotic liver tissue, suggesting FABP7-PPARγ as a therapeutic target [35]. Based on this body of evidence, further studies are warranted to determine whether analogous FABP7-dependent metabolic and immunoregulatory mechanisms operate in glioma, where FABP7 may similarly shape the immune microenvironment and promote tumor progression.

### 4.4. FABP7-Enriched Tumor Microtubes Modulate Neurocellular Networks to Promote Glioma Progression

A particularly novel aspect of GBM pathobiology involves the interactions between tumor cells and neurons mediated by FABP7. Recent studies have revealed that GBM cells form ultra-long membrane structures called tumor microtubes (TMs) that facilitate invasion and resistance through gap junction-mediated calcium redistribution (protecting against therapy-induced stress), nuclear and organelle transport for migration and self-repair, and glutamatergic synaptic integration via α-amino-3-hydroxy-5-methyl-4-isoxazolepropionic acid (AMPA) receptors that drives calcium-dependent proliferation [91]. Interestingly, FABP7 has recently emerged as a critical structural and signaling component driving TM formation. Using patient-derived GBM neurosphere cultures, Choi et al. (2025) demonstrated that FABP7, together with the axonal growth-associated protein GAP43, orchestrates TM biogenesis through an FABP7-protein kinase C (PKC)-GAP43 phosphorylation axis, enabling GBM cells to establish interconnected TM networks that sustain intercellular communication and promote directional infiltration into the brain [91]. Furthermore, genetic (siRNA-mediated) or pharmacological inhibition of FABP7 using SBFI-26, a small-molecule FABP7 inhibitor, was shown to disrupt TM network formation, impair GBM cell migration in vivo, and significantly prolong survival in orthotopic xenograft models [46]. This TM-mediated electrochemical coupling between glioma and neuronal networks has important implications for the TME and therapeutic resistance. Bidirectional interactions between GBM cells and neurons have been shown to increase neuronal hyperexcitability and seizure activity, while, conversely, neuronal activity promotes tumor growth [45,107,108,109,110]. Accordingly, glioma patients commonly experience seizures, and neural activity induced by glioma cells represents a key mechanism by which tumors remodel the brain milieu [111,112,113]. FABP7-enriched TMs may contribute to this pathological glioma–neuron network by potentially influencing both seizure susceptibility and immune surveillance, as seizures and neuroinflammation are closely linked in the brain through the activation of microglia and astrocytes that release pro-inflammatory cytokines, which, in turn, can lower seizure thresholds and facilitate tumor progression through immunosuppressive mechanisms [114]. In support of this hypothesis, our recent studies have shown that astrocytic FABP7 modulates nocturnal seizure threshold and activity-dependent gene and protein expression in the mouse brain [71,72]. In addition, we found that genetic deletion of *Fabp7* increases seizure resistance and alters immediate early gene induction and proteasomal responses following electroshock [71,72]. Thus, it is tempting to speculate that FABP7-mediated signaling in TMs may contribute to glioma-induced neuronal hyperexcitability and seizure generation, thereby representing a potential therapeutic target for disrupting tumor–neuron communication networks in GBM.

## 5. Discussion

The understanding of FABP7 has evolved from that of a developmentally enriched glial protein to a central regulator at the intersection of tumor metabolism, immune modulation, and neuronal network integration in glioma. Gliomas co-opt FABP7-dependent lipid trafficking and transcriptional programs to sustain stemness, metabolic adaptability, oxidative stress resilience, and invasive growth. Beyond its tumor-intrinsic functions, FABP7 also remodels the TME by activating immunosuppressive gene networks, promoting M2 polarization, limiting cytotoxic T-cell activity, and conferring resistance to T-cell-induced ferroptosis, thereby contributing to immune checkpoint resistance and adverse clinical outcomes. Emerging data further suggest that FABP7 links glioma progression to neural circuitry by coordinating synaptic regulation with TM-mediated neuron–glioma interactions. By controlling the convergence of ferroptosis resistance, immune evasion, and tumor microtube dynamics, FABP7 is uniquely positioned as a compelling, single-target candidate capable of addressing multiple axes of glioblastoma therapy resistance.

However, several critical research gaps remain. First, the post-transcriptional dimension of FABP7 regulation in cancer is largely unexplored. CPEB1 has been shown to function as a potent inducer of GSC differentiation [115] and glioblastoma cell migration [116], and can exhibit dualistic nature in either promoting or suppressing tumorigenesis [117]. Although CPEB1-mediated cytoplasmic polyadenylation of *Fabp7* has just begun to be characterized in normal astrocytes [63], whether this mechanism is dysregulated in GSCs, which share key developmental features with FABP7-expressing radial glia [47,48,49], has not been investigated. Post-transcriptional processing of *Fabp7* could also involve other RNA binding proteins (RBPs), such as CPEB4 [118,119], or in combination with recently identified differentially expressed RBPs (e.g., ELAVL2) in gliomas [120]. Given that alternative polyadenylation (APA) of transcripts is regulated over time-of-day and sleep homeostasis [72], and that APA landscapes influence tumor immune infiltration patterns, predict immunotherapy responses, and is globally altered in tumors [121,122,123], it will be important to determine whether *Fabp7* poly(A) dynamics or transcript isoform usage are disrupted in glioma. APA usage influences mRNA stability, localization, and local translation, and is increasingly being recognized as a process to regulate cell proliferation/differentiation, and regulating responses to environment [124], including immune-related genes in the TME in LGG [125]. The identification of a retroelement-driven chimeric *FABP7* transcript with distinct oncogenic properties in lymphoma [126] further supports the concept that aberrant mRNA processing can generate cancer-specific FABP7 isoforms and that FABP7 transcript diversity in glioma warrants systematic characterization. At the epitranscriptomic level, N^6^-methyladenosine (m6A) modification has emerged as a dominant post-transcriptional regulatory mechanism in GBM: loss of the m6A writer complex components methyltransferase-like 3 (METTL3) and methyltransferase-like 14 (METTL14) dramatically promotes GSC self-renewal and tumorigenesis, while inhibition of the m6A demethylase fat mass and obesity-associated protein (FTO) suppresses tumor growth and prolongs survival in GSC xenograft models, demonstrating that the m6A landscape is functionally critical for GSC biology [127]. Given that m6A modifications regulate mRNA stability, alternative splicing, and nuclear export of thousands of transcripts in glioma cells in a context-dependent manner [128,129], and that m6A readers such as insulin-like growth factor 2 mRNA-binding protein 2 (IGF2BP2) and insulin-like growth factor 2 mRNA-binding protein 3 (IGF2BP3) are themselves overexpressed in GBM where they stabilize pro-tumorigenic transcripts and regulate GSC ferroptosis resistance [130,131,132], it is plausible that the FABP7 transcript is itself subject to m6A-dependent stability regulation in GSCs, a possibility that could be interrogated through methylated RNA immunoprecipitation sequencing (meRIP-seq) in patient-derived GSC models. At the level of non-coding RNA regulation, a precedent for circular RNA (circRNA)-mediated post-transcriptional control of FABP family members in glioma already exists: circular RNA ATP-binding cassette subfamily B member 10 (circ-ABCB10) acts as a competing endogenous RNA (ceRNA) by sequestering microRNA-620 (miR-620), thereby relieving repression of FABP5 expression [133], and analogous circRNA or lncRNA sponging mechanisms targeting miRNAs with predicted binding sites in the *FABP7* 3′UTR may similarly operate in GBM but have not been characterized. Together, these considerations suggest that the post-transcriptional regulation of FABP7 in glioma encompasses a multi-layered network involving RBP-mediated mRNA stabilization, epitranscriptomic m6A modification, and non-coding RNA-mediated control that likely intersect with the alternative polyadenylation and transcript diversity mechanisms already discussed and that systematic characterization of this regulatory network through integrative enhanced crosslinking and immunoprecipitation sequencing (eCLIP-seq), meRIP-seq, and long-read isoform sequencing in GSC models will be essential for fully understanding how FABP7 expression becomes dysregulated during gliomagenesis.

Second, the mechanisms underlying FABP7’s immunomodulatory functions require further clarification. Although accumulating evidence supports a role for FABP7 in shaping a pro-tumorigenic TME, it remains unclear to what extent and through which mechanisms host/systemic FABP7 and tumor cell-derived FABP7 contribute to glioma progression, survival, and therapeutic responses. Orthotopic implantation of control versus Fabp7-deficient glioma cells into wild-type (WT) and *Fabp7* KO mice would help disentangle these contributions. Comparative immune phenotyping and single-cell transcriptomic analyses of both tumor and non-tumor compartments, including stromal and immune cells, could further elucidate cell type-specific mechanisms. Such approaches could elucidate FABP7-dependent signaling pathways and identify novel, cell type-specific neurotrophic and immunomodulatory biomarkers associated with disease progression and treatment response.

Third, the role of FABP7 in neuron–glioma interactions remains insufficiently defined. It has yet to be determined how host- or tumor cell-intrinsic FABP7 influences synapse formation and neuron–glioma connectivity. As outlined above, the combinatorial use of control and *Fabp7*-deficient glioma cells in WT and *Fabp7* KO mouse models, together with in vitro neuron–glioma co-culture systems [134] and in vivo paradigms that manipulate neuronal activity, such as optogenetic circuit stimulation [109] or electroshock-induced seizures [71,72], in the presence or absence of FABP7 could provide mechanistic insights into this neuron–astrocyte–tumor axis. Such approaches would generate direct causal evidence linking epilepsy-related hyperexcitability to glioma progression and clarify the specific contribution of FABP7 to this bidirectional pathogenic interaction.

Fourth, circadian biology may inform the optimal timing of FABP7-targeted and conventional therapies. Because FABP7 mRNA processing, protein abundance, and downstream signaling exhibit circadian oscillations [58,61,62], and blood–brain barrier (BBB) dynamics likely fluctuate with sleep/wake and circadian states [135,136,137] are regulated by FABP7 [73,138,139], the efficacy of FABP7-targeted therapies, and potentially ICB (e.g., PD-L1, CTLA-4) responses in FABP7-high tumors, may vary by time of day and BBB permeability. This hypothesis is supported by growing evidence that circadian timing influences chemotherapeutic efficacy, anti-tumor immunity, and immunotherapy responses [140,141,142,143,144,145].

Finally, clinical translation of FABP7-centered therapies will need to address safety. FABP7 is expressed in healthy astrocytes and neural progenitors, so systemic inhibition might have adverse effects on CNS functions such as sleep regulation and cognition. Consistent with this, the transient nature of FABP7-dependent myelination deficits and the dispensability of FABP7 for remyelination suggest that adult myelin maintenance would be preserved under therapeutic FABP7 inhibition [54]. Encouragingly, FABP7 inhibitors, such as MF6 and SBFI-26, have not been reported to exhibit overt toxicity in multiple sclerosis or orthotopic glioma xenograft models and have been associated with improved pathological outcomes [91,146]. A critical consideration for the clinical translation of FABP7 inhibitors concerns their BBB permeability and pharmacological suitability as CNS-active agents. MF6, a potent FABP7/FABP5 inhibitor (KD ≈ 20 nM and 874 nM, respectively), exhibits favorable brain penetration and oral bioavailability, achieving neuroprotective and anti-inflammatory effects in multiple preclinical CNS disease models without overt toxicity [146,147,148]. SBFI-26, a competitive FABP5/FABP7 inhibitor (Ki ≈ 0.9 μM and 0.4 μM, respectively), has demonstrated anti-tumor activity in preclinical cancer models, although its CNS pharmacokinetic profile remains less well characterized [149,150]. Potential mechanisms of resistance to FABP7-targeted therapies may include compensatory upregulation of alternative lipid transport pathways (e.g., FABP5 and CD36), metabolic reprogramming, and the substantial intratumoral heterogeneity of GBM, which may permit the persistence of FABP7-independent tumor cell populations [151]. These observations support the development of combination strategies integrating FABP7 inhibition with immunotherapeutic or metabolic-targeting approaches. The use of local delivery strategies, including nanoparticle-based formulations capable of co-delivering FABP7 inhibitors with ICBs via convection-enhanced delivery or implantable polymers, could also maximize therapeutic efficacy while minimizing systemic exposure and off-target effects [152,153,154].

## 6. Conclusions

In summary, FABP7 represents a glia-derived integrator of metabolic, circadian, and neuro–immune signaling networks that are repurposed in glioma to sustain tumor progression and therapeutic resistance. Targeting FABP7-centered pathways offers a conceptual strategy to simultaneously disrupt metabolic adaptation, restore immune surveillance, and interfere with tumor–neuron connectivity. Nevertheless, several challenges must be addressed before FABP7-targeted therapies can be translated into clinical practice. Current evidence is derived largely from preclinical studies, many of which do not fully recapitulate the molecular heterogeneity, tumor–immune interactions, and BBB complexity of human GBM. In addition, although emerging FABP7 inhibitors such as MF6 and SBFI-26 have shown encouraging efficacy and safety profiles in experimental models, further optimization of BBB penetration, pharmacokinetic properties, and target selectivity will be important to maximize therapeutic benefit while minimizing potential off-target effects in normal neural tissues. The absence of validated predictive biomarkers and clinical trial data also remains a significant barrier to patient stratification and therapeutic development. Future interdisciplinary research bridging cancer neuroscience, tumor immunology, chronobiology, and nano-biomedicine will be essential to systematically exploit FABP7-centered vulnerabilities and translate these insights into effective therapeutic strategies for GBM, the most treatment-resistant primary brain cancer.

## Figures and Tables

**Table 1:** FABP7 as a multifunctional oncogenic driver in glioma: biological functions, mechanistic roles, and therapeutic implications.

Biological Axis	FABP7 Function	Mechanistic Basis	Pathological Consequence Glioma	Therapeutic Implications	Key References
Lipid Metabolism and Stemness	Promotes fatty acid trafficking and lipid droplet formation	PUFA binding (AA, DHA); lipid droplet assembly; metabolic buffering	Enhances GSC maintenance and metabolic adaptation	FABP7 inhibitors (MF6, SBFI-26); metabolic targeting	[23,24,27,55,81]
Oncogenic Signaling Networks	Activates pro-tumorigenic pathways	Modulates Src, MEK/ERK, NF-κB, Wnt/β-catenin pathways	Increased proliferation, invasion, stem-like phenotype	Combination with pathway-specific inhibitors	[82,83,84,85,86]
Nuclear Transcriptional Regulation	Lipid-dependent transcriptional co-regulator	Nuclear translocation via NLS; interaction with PPARγ, RXRα, ACLY	Associated with EGFR amplification, IDH1-WT GBM; EMT and SOX2 activation	Target nuclear localization or FABP7–ACLY axis	[25,33,34,87,88,89,90]
Circadian Regulation	Acts as a glial clock integrator	BMAL1 and REV-ERBα regulation; rhythmic mRNA trafficking	Links metabolism to ferroptosis resistance and tumor survival	Chronotherapy strategies; time-of-day optimization	[36,58,60,61,62,63]
Immunosuppressive TME Modulation	Shapes cold immune microenvironment	Upregulates Treg/MDSC genes; reduces CD8^+^ T-cell infiltration	Immune evasion; poor prognosis	Combine FABP7 inhibition with PD-1/PD-L1 blockade	[26,36]
Ferroptosis Resistance	Protects against T-cell–induced lipid peroxidation	Suppresses LPCAT3; enriches MUFAs/TAGs; BMAL1 Upregulation	Immune checkpoint resistance	Combine ferroptosis induction with immunotherapy	[36]
Macrophage Polarization	Promotes M2-like TAM phenotype	FABP7–PPARγ signaling; lipid metabolic reprogramming	Pro-tumoral macrophage activation; immune suppression	Target FABP7–PPARγ axis	[35,37]
Tumor Microtubes and Neuron–Glioma Crosstalk	Drives tumor microtube formation and synaptic integration	FABP7–PKC–GAP43 axis	Enhanced invasion, neuronal hyperexcitability, seizure susceptibility	TM disruption; FABP7 inhibition (SBFI-26)	[91]

Abbreviations: AA, Arachidonic Acid; ACLY, ATP-Citrate Lyase; BMAL1, Brain and Muscle ARNT-Like 1; CD8, Cluster of Differentiation 8; DHA, Docosahexaenoic Acid; EGFR, Epidermal Growth Factor Receptor; EMT, Epithelial–Mesenchymal Transition; ERK, Extracellular Signal-Regulated Kinase; FABP7, Fatty Acid-Binding Protein 7; GAP43, Growth-Associated Protein 43; GBM, Glioblastoma; GSC, Glioma Stem Cell; IDH1-WT, Isocitrate Dehydrogenase 1 Wild-Type; LPCAT3, Lysophosphatidylcholine Acyltransferase 3; MEK, Mitogen-Activated Protein Kinase Kinase; MDSC, Myeloid-Derived Suppressor Cell; MF6, Small-Molecule FABP7 Inhibitor MF6; MUFAs, Monounsaturated Fatty Acids; NF-κB, Nuclear Factor Kappa B; NLS, Nuclear Localization Signal; PD-1, Programmed Cell Death Protein 1; PD-L1, Programmed Death-Ligand 1; PKC, Protein Kinase C; PPARγ, Peroxisome Proliferator-Activated Receptor Gamma; PUFA, Polyunsaturated Fatty Acid; REV-ERBα, Nuclear Receptor Subfamily 1 Group D Member 1; RXRα, Retinoid X Receptor Alpha; SBFI-26, Small-Molecule FABP7 Inhibitor 26; SOX2, SRY-Box Transcription Factor 2; Src, Proto-Oncogene Tyrosine-Protein Kinase Src; TAGs, Triacylglycerols; TAM, Tumor-Associated Macrophage; TME, Tumor Microenvironment; Treg, Regulatory T-Cell; Wnt, Wingless/Integrated Signaling Pathway.

## Data Availability

No new data were created or analyzed in this study.

## Abbreviations

The following abbreviations are used in this manuscript:

AA	Arachidonic Acid
ACLY	ATP-Citrate Lyase
AD	Alzheimer’s Disease
ALS	Amyotrophic Lateral Sclerosis
AMPA	α-Amino-3-hydroxy-5-methyl-4-isoxazolepropionic Acid
APA	Alternative Polyadenylation
ARNTL	Aryl Hydrocarbon Receptor Nuclear Translocator-Like Protein 1
B-FABP	Brain-Type Fatty Acid-Binding Protein
BDNF	Brain-Derived Neurotrophic Factor
BMAL1	Brain and Muscle ARNT-Like 1
BLBP	Brain Lipid-Binding Protein
CAFs	Cancer-Associated Fibroblasts
CNS	Central Nervous System
CLOCK	Circadian Locomotor Output Cycles Kaput
Col5a1	Collagen Type V Alpha 1 Chain
CPE	Cytoplasmic Polyadenylation Element
CPEB1	Cytoplasmic Polyadenylation Element Binding Protein 1
CRY	Cryptochrome
CSC	Cancer Stem Cell
DHA	Docosahexaenoic Acid
EGFR	Epidermal Growth Factor Receptor
EMT	Epithelial–Mesenchymal Transition
Eno1	Enolase 1
FA	Fatty Acid
FABP7	Fatty Acid-Binding Protein 7
GBM	Glioblastoma
GSC	Glioma Stem-Like Cell
ICB	Immune Checkpoint Blockade
IDH	Isocitrate Dehydrogenase
Il11	Interleukin 11
KO	Knockout
LD	Lipid Droplet
LGG	Lower-Grade Glioma
LPCAT3	Lysophosphatidylcholine Acyltransferase 3
MDSC	Myeloid-Derived Suppressor Cell
MEK	Mitogen-Activated Protein Kinase Kinase
MUFAs	Monounsaturated Fatty Acids
NF-κB	Nuclear Factor Kappa-Light-Chain-Enhancer of Activated B-Cells
NLS	Nuclear Localization Signal
NR1D1/2	Nuclear Receptor Subfamily 1 Group D Members 1 and 2
NSC	Neural Stem Cell
NPC	Neural Progenitor Cell
OPC	Oligodendrocyte Progenitor Cell
OV	Overexpression
PAP	Peripheral Astrocytic Process
PD-1	Programmed Cell Death Protein 1
PD-L1	Programmed Death-Ligand 1
PI3K	Phosphoinositide 3-Kinase
PKC	Protein Kinase C
PML NB	Promyelocytic Leukemia Nuclear Body
PPARγ	Peroxisome Proliferator-Activated Receptor Gamma
PTN	Pleiotrophin
PUFA	Polyunsaturated Fatty Acid
REV-ERB	Reverse Erythroblastosis Virus Alpha
RORE	ROR Response Element
ROR	RAR-Related Orphan Receptor
ROS	Reactive Oxygen Species
RXRα	Retinoid X Receptor Alpha
shRNA	Short Hairpin RNA
SOX2	SRY-Box Transcription Factor 2
Src	Proto-Oncogene Tyrosine-Protein Kinase Src
TAM	Tumor-Associated Macrophage
TCGA	The Cancer Genome Atlas
TGF-β	Transforming Growth Factor Beta
TME	Tumor Microenvironment
TM	Tumor Microtube
Treg	Regulatory T-Cell
WT	Wild-Type
WHO	World Health Organization
ZEB1	Zinc Finger E-Box Binding Homeobox 1
